# Prediction of rehospitalization and mortality risks for skilled nursing facilities using a dimension reduction approach

**DOI:** 10.1186/s12877-023-03995-y

**Published:** 2023-06-28

**Authors:** Juan Camilo David Gomez, Amy Cochran, Maureen Smith, Gabriel Zayas-Cabán

**Affiliations:** 1 Department of Industrial and Systems Engineering, University of Wisconsin-Madison, Madison, USA; 2 Department of Population Health Sciences, Department of Mathematics, University of Wisconsin-Madison, Madison, USA; 3 Department of Industrial and Systems Engineering and BerbeeWalsh Department of Emergency Medicine, University of Wisconsin-Madison, 3107 Mechanical Engineering Building, 1513 University Avenue, Madison, WI 53726 USA

**Keywords:** Skilled nursing facility, Rehospitalization, Factor analysis, Cluster analysis, GEE regression.

## Abstract

**Background:**

Hospitals are incentivized to reduce rehospitalization rates, creating an emphasis on skilled nursing facilities (SNFs) for post-hospital discharge. How rehospitalization rates vary depending on patient and SNF characteristics is not well understood, in part because these characteristics are high-dimensional. We sought to estimate rehospitalization and mortality risks by patient and skilled nursing facility (SNF) leveraging high-dimensional characteristics.

**Methods:**

Using 1,060,337 discharges from 13,708 SNFs of Medicare patients residing or visiting a provider in Wisconsin, Iowa, and Illinois, factor analysis was performed to reduce the number of patient and SNF characteristics. K-means clustering was applied to SNF factors to categorize SNFs into groups. Rehospitalization and mortality risks within 60 days of discharge was estimated by SNF group for various values of patient factors.

**Results:**

Patient and SNF characteristics (616 in total) were reduced to 12 patient factors and 4 SNF groups. Patient factors reflected broad conditions. SNF groups differed in beds and staff capacity, off-site services, and physical and occupational therapy capacity; and in mortality and rehospitalization rates for some patients. Patients with cardiac, orthopedic, and neuropsychiatric conditions are associated with better outcomes when assigned to SNFs with greater on-site capacity (i.e. beds, staff, physical and occupational therapy), whereas patients with conditions related to cancer or chronic renal failure are associated with better outcomes when assigned to SNFs with less on-site capacity.

**Conclusions:**

Risks of rehospitalization and mortality appear to vary significantly by patient and SNF, with certain SNFs being better suited for some patient conditions over others.

**Supplementary Information:**

The online version contains supplementary material available at 10.1186/s12877-023-03995-y.

## Background

How hospitals can improve the management of patient care after discharge has become a national focus in the US in recent years [1]. Prior to the enactment of the Patient Protection and Affordable Care Act (or Affordable Care Act), when traditional fee-for-service reimbursement was common, hospitals had few financial incentives to curtail potentially high readmission rates [2]. After the enactment of the Affordable Care Act, many hospitals became increasingly liable for patient outcomes after hospitalization [3, 4] via a mixture of (i) changes in the way hospital are reimbursed from fee-for-service to value-based purchasing and bundled payments, (ii) changes in how doctors, hospitals, and other healthcare providers are able to organize through Accountable Care Organizations, and (iii) through penalties on hospitals with high readmission rates [2, 4, 5]. One result has been that hospitals have turned their focus to the process of how patients are assigned to post-acute facilities, such as skilled nursing facilities (SNFs), which provide short-term rehabilitative services after hospital discharge, as a potential strategy for reducing high rates of readmissions. This paper investigates how the assignment to a SNF after hospitalization impacts rehospitalization and mortality risk among individuals who have a Medicare claim for visiting a SNF after hospitalization and who reside in Wisconsin, Illinois, or Iowa, or have a visit to any provider in these states.

SNFs are a frequent post-acute care destination for many patients in the US: they account for more than 40% of transfers to a post-acute facility after discharge, [6] with roughly 20% of Medicare beneficiaries transferred to a SNF after hospitalization [7]. It has been documented that the rates of readmission associated with SNFs are both high and variable, [3, 8–11] with roughly 25% of patients discharged to a SNF requiring rehospitalization within 30 days, and a majority of these rehospitalizations have been deemed as preventable [8, 12].

One way to reduce readmissions is to transfer patients to the most appropriate SNF since some SNFs are better able to provide skilled care to certain patients than others [5, 13, 14]. Further, SNFs vary greatly in the resources available to patients, including the number of staff by fulltime versus parttime, on-site versus off-site, or specialty (e.g., mental health, speech pathology, physical therapy). As a result, SNFs vary greatly in overall hospital readmission rates [15]. This has motivated policy makers to use hospital readmission rates to evaluate the quality of care at a SNF [16].

Because readmission rates may differ by SNF and patient characteristics, an overall readmission measure may mask underlying variation in SNF performance by patient characteristics [17–19]. The extent of variation in readmission rates among SNFs by different characteristics is often unknown but, if significant, suggests that SNFs with very low readmission rates for certain patients and very high readmission rates for others will have their actual quality of care masked.

This study examines whether certain SNFs characterized by the type of providers and services they offer differed in their readmission rates by patient characteristics of the initial hospitalization. We expected that one SNF may be better suited for specific comorbidities or diagnoses over another. We also expected that the patient population of one SNF would differ from that of another. If these differences exist, then it is important to disentangle the contribution that the SNF itself has to quality of care measures from the contribution that the patient population has to these measures, thus motivating our efforts to evaluate SNFs by patient characteristics. For one example, it is recommended that individuals receive rehabilitation after a cardiac event with components specific to cardiac patients [20, 21]. Therefore, individuals after a cardiac event may be more likely to be discharged to a SNF with services that can provide this type of rehabilitation and receive better care than a SNF without these services. Further, it may also yield insight into the specific SNF characteristics that make it more appropriate for a patient with a particular set of characteristics. In short, we expect that this analysis will contribute to our understanding of whether to discharge a patient with specific characteristics to SNF with certain characteristics.

Our sample population consists of individuals who had Medicare Part A and Part B insurance and who were either residents of three states (Wisconsin, Illinois, or Iowa) or had visits to any provider in one of these states. Using Medicare claims data, we analyzed 1,060,337 discharges from 639,373 unique patients assigned to 13,708 SNFs. We used factor analysis to reduce the number of clinical variables on each patient and each SNF into a more manageable number of patient and SNF factors. We next placed SNFs into distinct groups based on their factor scores. We then modeled the risk of rehospitalization and the risk of death within 60 days by the resulting patient and SNF characteristics. We used unadjusted and adjusted generalized estimating equations logistic regression models to estimate these risks across SNFs and patients.

## Methods

### Data

The data consists of claims and enrollment files from Medicare patients with fee-for-service and with Part A and Part B insurance. Claims were included when the patient was (i) admitted to an acute care hospital and discharged to a SNF, and (ii) was a resident of Wisconsin during 2010–2017, Illinois during 2014–2017, and Iowa during 2016–2017 or made a claim for *any* services provided in these states in the given time period. All Medicare patients were included, regardless of the original reason for Medicare eligibility. Claims were excluded when the patient was enrolled in a Health Maintenance Organization or had railroad benefits. Additional claims were excluded when SNF care was a swing bed (59,451 claims) [22] and when SNF variables were missing (1,974 claims) or corrupted (47 claims). Claims from patients who die during the time frame were included if they otherwise met inclusion and exclusion criteria. The final sample was 1,060,337 SNF discharges across 13,708 SNFs. Although these 13,708 SNFs are located across the country, SNFs in Wisconsin, Iowa, or Illinois account for a large proportion (74%) of the SNF discharges.

### Variables

Variables were identified at patient (Table 1) and SNF levels (Table 2). Patient variables include age, gender, race/ethnicity, insurance coverage, residence state, hospital length of stay, comorbidity conditions (76 conditions), and Diagnostic Related Group (DRG) codes associated with each hospital stay. Race and ethnicity in claims data is defined by first sourcing information from the Social Security Administration and then applying an algorithm from the Centers for Medicare & Medicaid Services, resulting in 7 categories (Asian/Pacific Islander, Black, Hispanic, Native American, Other, White, and Unknown). DRG codes contain information on diagnosis and accompanying complications. In the factor analysis, we only analyzed information related to diagnosis and not whether there were complications, leaving 340 codes for analysis. An indicator of complication was later added to regression models. No patient variables were missing in the data. SNF variables include types of providers, number of beds and specialty beds (14 variables), number of fulltime or parttime nurses/specialists (93 variables), and indicators for off-site and in-site services offered at SNF (46 variables). Each claim had variables related to the assigned SNF, which took on the same value for every claim associated with a given SNF, allowing us to extract SNF variables in the dataset. The two outcomes available in the data are rehospitalization within 60 days of discharge from the SNF and mortality within 60 days of discharge from the hospital.

**Table 1:** Sample statistics of claims (n = 1,060,337)

Variable name	Value
Outcomes	
Death within 60 days of hospital discharge, n (%)	139,452 (13.1)
60 days Rehospitalization, n (%)	320,726 (30.2)
Patient variables	
Hospital length of stay in days, mean (SD)	6.2 (5.0)
Age in years, mean (SD)	78.5 (9.9)
Female, n (%)	664,367 (62.6)
Complicated, n (%)	678,979 (64.0)
Race/ethnicity, n (%)	
- Asian/Pacific islander	6245 (0.5)
- Black	89,364 (8.4)
- Hispanic	8732 (0.8)
- Native American	2999 (0.3)
- Other	7126 (0.7)
- Unknown	2974 (0.3)
- White	942,897 (88.9)
Comorbidities (top 5), n (%)	
- Hypertension	754,461 (71.1)
- Hyperlipidemia	502,358 (47.3)
- Osteoarthritis	379,716 (35.8)
- Diabetes mellitus	352,410 (33.2)
- Conduction disorder or cardiac dysrhythmia	309,973 (29.2)
DRGs (top 5), n (%)	
- Major hip and knee joint replacement or Reattachment of lower extremity	89,830 (8.4)
- Septicemia or severe sepsis	79,700 (7.5)
- Hip and femur procedures except major joint	46,116 (4.3)
- Simple pneumonia and pleurisy	39,914 (3.7)
- Kidney and urinary tract infections	38,000 (3.5)

**Table 2:** Sample statistics of SNFs (n = 13,708)

Variable name	Value
Number of beds (top 5), mean (SD)	
- Total number of beds	111.4 (61.4)
- Medicare/Medicaid beds	109.3 (60.3)
- Dually certified beds	100.3 (63.6)
- Number of beds - Alzheimer	5.0 (14.2)
- Number of beds - Rehabilitation	1.0 (6.8)
Staff count (top 5), mean (SD)	
- Certified nurse aides - full time	33.1 (25.7)
- Licensed practical/vocational nurses - full time	12.6 (12.9)
- Food service personnel - full time	8.8 (8.2)
- Certified nurse aides - part time	7.1 (10.1)
- Housekeeping personnel - full time	6.9 (10.5)
Services (top 5), n (%)	
- Nursing services on-site	13,680 (99.8)
- Dietary services on-site	13,661 (99.6)
- Housekeeping services on-site	13,630 (99.4)
- Physical Therapy on-site	13,604 (99.2)
- Occupational therapy on-site	13,588 (99.1)
Provider type, n (%)	
- Skilled Nursing Facility/Nursing Facility (Dually Certified)	10,811 (78.8)
- Skilled Nursing Facility/Nursing Facility (Distinct Part)	2095 (15.2)
- Skilled Nursing Facility	802 (5.8)
Urban indicator, n (%)	
- Urban	10,111 (73.7)
- Rural	3597 (26.3)

### Analysis

Variables were analyzed in three steps. First, exploratory factor analysis reduced the dimension of patient variables and separately, SNF variables [23–25]. Second, K-means clustering was applied to SNF factor scores to categorize SNFs into groups [26]. Third, we estimated unadjusted and adjusted risks of rehospitalization and death within 60 days of discharge by patient factor and SNF cluster using logistic regression models.

#### Dimension reduction

Factor analysis was first applied to DRG codes and comorbidities (416 variables). Since a patient may have multiple claims for SNF visits, we first averaged the binary indicators of DRG codes and comorbidities over each person’s set of claims available in the data, which consisted of the final sample of 1,060,337 claims meeting inclusion and exclusion criteria and the additional 61,472 claims excluded because SNF care was a swing bed or SNF variables were missing or corrupted. We then applied factor analysis to the averaged indicators. Factor analysis was then applied to all SNF variables (154 variables).

Factor analysis was performed using the R package *psych.* [27] The varimax rotation was selected. The number of latent factors was chosen to balance interpretability and model fit. Model fit was measured with the Very Simple Structure (VSS) score [28]. An output of performing factor analysis is a matrix of factor loadings, which we used to interpret factors. An entry in the loading matrix quantifies the degree to which a variable is an indicator of an underlying factor. Factor models were used to assign Thurstone factor scores [24, 29] to each claim and each SNF. SNF factor scores were used in the categorization step, and patient factor scores were used directly in the regression model. Even though the factor model was built using variables averaged over a single patient’s visits, we assigned factor scores to each visit based on variables for that visit. This is important, since in practice, a patient would be assigned factor scores at a visit based on current DRG codes and comorbid conditions and then moved to a SNF that provides good care for patients with similar factor scores.

#### Categorization

Next, we categorized SNFs into groups based on factor scores. This facilitates the use of SNF factor scores in practice, since it is easier to evaluate the impact of sending a patient to SNF group than evaluate scores directly. SNFs were categorized by applying K-means clustering to SNF factor scores. Categorization was implemented in the Python library Scikit-learn [30]. The number of groups was chosen to maximize mean silhouette score. Silhouette score measures similarity of a SNF to SNFs in the same group relative to SNFs in other groups. Similarity is measured using the Euclidean norm. After selecting the number of groups, we assigned each SNF to the group whose centroid is closest to this SNF’s factor scores.

#### Risk prediction

Our final analyses estimate risks of rehospitalization and death. Logistic regression models were built to recover unadjusted and adjusted estimates using the generalized estimating equation (GEE) framework [31]. The GEE framework allows for correlations due to multiple claims from the same individual from the same state. Particularly, we specified a covariance structure which was the sum of two covariance matrices with compound symmetry. The first matrix captured a fixed correlation between visits from the same state. The second captured a fixed correlation between visits from the same person.

Each regression model included independent variables recovered from dimension reduction and categorization steps: patient factors scores and the assigned SNF group. Interactions between each factor score and each SNF group were also included. The dependent variable was either rehospitalization or death within 60 days. No other variables were included, upon which *unadjusted* risk estimates could be recovered conditional on the person having a certain set of factor scores and being assigned to a specific SNF group. Mean centered control variables (age, race/ethnicity, gender, complications, and hospital length of stay) were then added to the models to recover *adjusted* versions of our estimates. We report estimates of adjusted and unadjusted *risks* of mortality and readmission were a certain patient assigned to a given SNF, marginalized over other covariates. These risks were obtained by averaging model predicted risks among visits with a given patient factor score surpassing + 1 standard deviation from its mean [32]. Finally, we perform joint Wald hypothesis tests to investigate whether the risk of a given dependent variable is equal across SNFs for a person with given factor scores and covariates. Significance was *p* < 0.05.

Additional details and analyses are reported in Additional File 1. These include unadjusted and adjusted *odds*, which are common quantities to report but left out of the main text due to difficulty in comparing odds between studies and models (Additional Tables A1-A4), [32, 33] and a check on sensitivity of adjusted risk estimates to time of discharge (Additional Tables A5-A6). We also provide additional information on model fits, including VSS scores, model accuracy, and variables that cross-load on more than one factor (Additional Table A7-A10).

## Results

### Dimension reduction

**Fig. 1:**
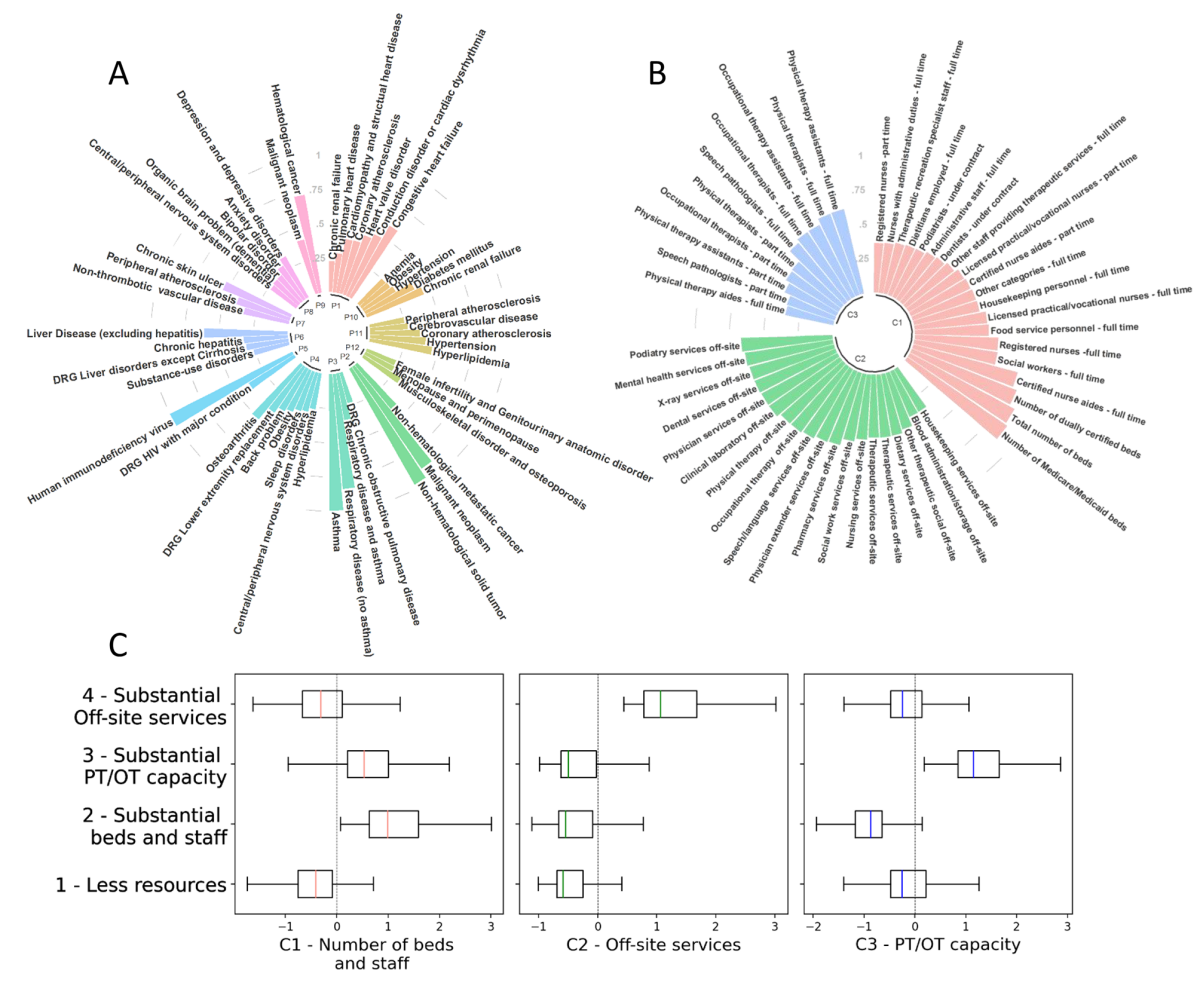
**A)** Factor loadings (exceeding 0.2) for patient variables and patient factors. **B)** Factor loadings (exceeding 0.2) for SNF variables and facility factors. **C)** Distribution of SNF factor scores per SNF group

Our analysis suggests 12 latent factors explain variation among patient comorbidities and DRGs (Additional Table A11). These factors reflect broad sets of common conditions and diagnoses. For readability, each factor is labeled P1 to P12 and referenced together with the variable that loaded most strongly onto the factor, which are, respectively, (P1) congestive heart failure, (P2) non-hematological solid tumor, (P3) asthma, (P4) osteoarthritis, (P5) human immunodeficiency virus (HIV), (P6) liver disease (excluding hepatitis), (P7) chronic skin ulcer, (P8) depression and depressive disorders, (P9) hematological cancer, (P10) chronic renal failure, (P11) hyperlipidemia, and (P12) other musculoskeletal including osteoporosis. Factors are interpreted in terms of the variables that load most strongly on each factor, which are depicted in Fig. 1A.

Our analysis suggests three factors describe the variability among SNF variables. These factors correspond to SNF capacity and services provided. They were labelled C1 to C3 and interpreted as number of beds and staff, off-site services (e.g., podiatry, mental health, x-ray, dental services) and physical therapy (PT) and occupational (OT) therapy capacity. Figure 1B shows factor loadings for SNF variables.

### Categorization

Upon applying K-means clustering to SNF factor scores, a 4 group solution was selected, which gave the highest average silhouette score for the 3 factor solution (Additional Figure S1). SNF Group 1 was largest with 51.4% of SNFs, followed by SNF Group 4 with 20.8% of SNFs, SNF Group 3 with 15.8% of SNFs, and SNF Group 2 with 11.8% of SNFs.

Driving factors for categories were number of beds and staff, off-site services, or number of PT/OT staff. Figure 1 C shows the distribution of factor scores per group for the 4 group solution. SNF Group 2 had a considerable number of beds and specialists, as indicated by an interquartile range above zero for C1 factor scores. SNF Group 4 was marked for providing considerable off-site services (factor C2). SNF Group 3 was marked for providing considerable PT/OT staff and number of beds (factors C3 and C1). Remaining SNFs made up SNF Group 1, which had less resources as indicated by interquartile range below or crossing zero for all the factors. For summary statistics of claims and SNFs by SNF group, see Additional Tables A12-A13.

### Rehospitalization risk

We investigated whether certain SNF groups had lower risks of rehospitalization for patients with a given set of factor scores. Adjusted risk of rehospitalization is provided in Table 3. When interpreting these risks, it is worth noting that, while Table 3 presents estimated risks for patients who score highly on the given factor, some patients may score highly on more than one factor. Among the 12 types of patients considered, corresponding to the 12 patient factors, we found significantly lower adjusted risk of rehospitalization in certain SNF groups for four patient types. These types were patients with a large score for P2 (non-hematological solid tumor), P4 (osteoarthritis), P8 (depression and depressive disorders), or P10 (chronic renal failure). These four patient types were also flagged as having significantly lower unadjusted rehospitalization risk (Additional Table A14) and adjusted rehospitalization risk after additionally controlling for discharge date (Additional Table A5). Assignment to a SNF group does not appear to greatly affect rehospitalization risk for the remaining types of patients.

**Table 3:** Adjusted risk, in percentages, of rehospitalization (95% confidence intervals) for each patient factor and SNF group

Patient factor	SNF group				
	1	2	3	4	*p*
1–Congestive heart failure	36.6 36.3–37.0	36.6 36.1–37.0	37.2 36.8–37.5	36.4 36.0-36.9	0.15
**2–Non-hematological solid tumor**	31.1 30.7–31.5	31.9 31.4–32.5	32.5 32.0-32.9	31.3 30.7–31.9	< 0.001
3–Asthma	33.9 33.6–34.2	33.9 33.4–34.3	33.8 33.4–34.2	34.0 33.5–34.4	0.98
**4–Osteoarthritis**	22.6 22.4–22.9	21.9 21.6–22.2	21.7 21.4–22.0	23.5 23.2–23.9	< 0.001
5–HIV	39.9 35.2–44.5	39.2 34.7–43.7	35.3 30.8–39.9	38.0 31.1–44.9	0.57
6–Liver disease	38.4 37.8–39.0	38.4 37.5–39.3	38.1 37.3–38.9	37.4 36.5–38.3	0.08
7–Chronic skin ulcer	36.0 35.7–36.3	35.6 35.2–36.1	36.0 35.6–36.4	35.7 35.2–36.2	0.66
**8–Depression and depressive disorders**	28.3 28.1–28.6	27.9 27.5–28.3	28.5 28.1–28.8	27.8 27.4–28.2	< 0.001
9–Hematological cancer	35.3 34.4–36.2	36.6 35.3–37.9	36.2 35.1–37.3	35.5 34.1–36.8	0.07
**10–Chronic renal failure**	35.5 35.2–35.7	35.7 35.3–36.1	36.1 35.7–36.4	35.1 34.7–35.5	< 0.001
11–Hyperlipidemia	31.6 31.3–31.8	31.3 30.9–31.6	31.4 31.1–31.7	31.3 30.9–31.6	0.24
12–Other musculoskeletal including osteoporosis	27.7 27.5–28.0	27.2 26.8–27.6	27.5 27.2–27.8	27.6 27.2–28.0	0.48

*Note*. SNF Group 1 = less resources; SNF Group 2 = substantial beds and staff; SNF Group 3 = substantial PT/OT staff; SNF Group 4 = substantial off-site services. Patient factors are bolded if the corresponding *p*-value is less than 0.05. Each patient factor is labeled by a number and the variable that loaded most strongly onto the factor

We expand on these differences. SNF Group 4 had lower rehospitalization rates for two of the four patient groups compared to the other SNFs. SNF Group 3 had the highest rehospitalization rates for three of the four patient groups. Recall, SNF Group 4 corresponds to SNFs with higher factor scores related to off-site services, and SNF Group 3 corresponds to SNFs with higher factor scores related to PT/OT capacity.

SNF Group 4 yielded the lowest rehospitalization rates for patients with depression and depressive disorders and correlated conditions (adjusted risk of 27.8% [95% CI: 27.4–28.2]) and chronic renal failure and correlated conditions (adjusted risk of 35.1% ([95% CI: 34.7–35.5]). Patients with osteoarthritis and correlated conditions had the lowest adjusted risks of rehospitalization of 21.7% (95% CI: 21.4–22.0) and 21.9% (95% CI: 21.6–22.2) when assigned to SNF Groups 3 and 2, respectively. Lastly, SNF Group 1 yielded the lowest rehospitalization rate (adjusted risk of 31.1% [95% CI: 30.7–31.5]) for patients with a non-hematological solid tumor and correlated conditions.

### Mortality risk

We investigated whether certain SNF groups had lower risks of mortality than others for patients with a given set of factor scores. Adjusted risks of mortality are provided in Table 4. Adjusted risks of mortality differed significantly across SNF groups for patients with a large score for patient factors P1 (congestive heart failure), P4 (osteoarthritis), P7 (chronic skin ulcer), and P8 (depression and depressive disorders). These four patient types were also flagged as having significantly lower unadjusted mortality risk (Additional Table A15) and adjusted mortality risk after additionally controlling for discharge date (Additional Table A6). Assignment to an SNF group does not appear to significantly affect mortality risk for the remaining types of patients.

**Table 4:** Adjusted risk, in percentages, of mortality (95% confidence intervals) for each patient factor and SNF cluster

Patient factor	SNF group				
	1	2	3	4	*p*
**1–Congestive heart failure**	20.2 19.9–20.5	18.8 18.4–19.2	19.2 18.8–19.5	20.2 19.7–20.6	< 0.001
2–Non-hematological solid tumor	20.9 20.5–21.2	20.8 20.2–21.3	21.1 20.7–21.6	21.2 20.6–21.8	0.40
3–Asthma	17.0 16.7–17.3	16.7 16.3–17.0	16.6 16.2–16.9	17.0 16.6–17.4	0.28
**4–Osteoarthritis**	5.0 4.9–5.1	5.2 5.1–5.3	5.3 5.2–5.4	5.0 4.9–5.2	< 0.001
5–HIV	11.4 8.1–14.6	11.6 8.4–14.7	13.7 10.2–17.2	13.2 7.9–18.5	0.73
6–Liver disease	17.8 17.3–18.3	17.1 16.4–17.8	17.2 16.5–17.8	17.7 16.9–18.5	0.29
**7–Chronic skin ulcer**	15.6 15.4–15.9	16.4 16.0-16.7	15.5 15.2–15.8	16.2 15.8–16.6	< 0.001
**8–Depression and depressive disorders**	11.1 10.9–11.3	10.6 10.4–10.9	10.9 10.6–11.1	11.2 10.9–11.5	0.007
9–Hematological cancer	21.6 20.7–22.4	21.3 20.1–22.5	21.2 20.2–22.3	22.1 20.7–23.4	0.82
10–Chronic renal failure	15.8 15.5–16.0	15.9 15.5–16.2	15.5 15.2–15.8	15.9 15.6–16.3	0.21
11–Hyperlipidemia	13.7 13.5–13.9	13.7 13.4–14.0	13.6 13.3–13.8	13.9 13.6–14.2	0.81
12–Other musculoskeletal including osteoporosis	11.2 11.0-11.4	11.2 11.0-11.5	11.3 11.0-11.5	11.2 10.9–11.5	0.63

*Note*. SNF Group 1 = less resources; SNF Group 2 = substantial beds and staff; SNF Group 3 = substantial PT/OT staff; SNF Group 4 = substantial off-site services. Patient factors are bolded if the corresponding *p*-value is less than 0.05. Each patient factor is labeled by a number and the variable that loaded most strongly onto the factor

For patients with congestive heart failure (and correlated conditions) or depression and depressive disorders (and correlated conditions), SNF Group 2 yielded the lowest adjusted mortality risk: respectively, 18.8% (95% CI: 18.4–19.2) and 10.6% (95% CI: 10.4–10.9). In contrast, SNF Groups 1 (adjusted risk of 5.0% [95% CI: 4.9–5.1]) and 4 (adjusted risk of 5.0% [95% CI: 4.9–5.2)]) yielded the lowest adjusted mortality risk for patients with osteoarthritis (and correlated conditions), whereas the SNF Group 3 yielded the lowest adjusted mortality risk for patients with chronic skin ulcer (and correlated conditions; adjusted risk of 15.5% [95% CI: 15.2–15.8]).

## Discussion

Using Medicare claims records (n = 1,060,337), this paper investigates how to discern SNFs with lower rehospitalization and mortality rates for a patient based on clinical variables (416 patient variables and 154 SNF variables). We proposed factor analysis models that reduce patient and SNF variables to a more manageable number of factors. Patient variables were reduced to 12 factors capturing broadly defined diseases or disorders, such as cancer, cardiac, or neuropsychiatric disorders. SNF variables were reduced to three factors capturing types and levels of services, facility capacity, and PT/OT capacity. Since it is more practical to assign patients to a SNF group than a SNF with certain factor scores, we went one step further after dimension reduction to categorize SNFs into four groups, differing on whether they had substantial off-site services, number of beds and specialists, or substantial PT/OT staff. We then estimated the risk of rehospitalization and of death within 60 days of hospital discharge for combinations of patient factor scores and SNF groups. This allowed us to identify SNF groups that have the lowest risks for various types of patients.

Our main finding is that SNF assignment can impact the risk of rehospitalization and mortality for some *but not all* patients. On one hand, it is encouraging to find that SNFs with varying levels of services and patient capacity can provide comparable outcomes for many patients. This may ease the mind of providers, patients, and family members when deciding which SNF to send a patient. On the other, it provides an opportunity to optimize SNF assignments for those patients for whom this assignment does matter. Moreover, the “best” SNF is not necessarily the same one for everyone.

Preliminary evidence is provided on *how* to assign SNFs for certain patients. Several types of patients appear to benefit from SNFs with more on-site resources. Cardiac patients – marked by congestive heart failure, conduction disorder or cardiac dysrhythmia, heart valve disorder, coronary atherosclerosis, cardiomyopathy, and pulmonary heart disease – were associated with a 1.8% lower mortality risk when assigned to SNFs with substantial beds and staff (SNF Group 2) when compared to SNFs with relatively fewer resources (SNF Group 1). Neuropsychiatric patients – marked by depression and depressive disorders, anxiety disorder, bipolar disorder, organic brain problem (dementia), central/peripheral nervous system disorders – also appear to benefit from a 0.4% lower rehospitalization risk and 0.5% lower mortality risk when assigned to SNFs with substantial beds and staff (SNF Group 2) compared to SNFs with relatively fewer resources (SNF Group 1). Orthopedic patients – marked by osteoarthritis, DRG lower extremity replacement, a back problem – had 1.8% lower rehospitalization risk when assigned to SNFs with substantial PT/OT staff (SNF Group 3) compared to SNFs with substantial off-site services but otherwise relatively fewer resources (SNF Group 4), although the lower rehospitalization risk was accompanied by a 0.3% increase in mortality risk. One possibility is that the resource rich SNF is able to provide the specialized or high intensity rehabilitation recommended to patients with cardiac, neuropsychiatric, or orthopedic conditions [20, 21, 34, 35].

Interestingly, other types of patients benefited from SNFs with fewer on-site resources. Patients with cancer were associated with lower rehospitalization risk, upwards of 1.4% lower, in the SNF with fewer resources (SNF Group 1) or only off-site resources (SNF Group 4) when compared with the SNFs with substantial beds and staffs (SNF Group 2) or PT/OT staff (SNF Group 3). Similarly, patients with chronic renal failure, diabetes mellitus, and correlated conditions were associated with lower rehospitalization risk, upwards of 1.0% lower, in the SNF with fewer resources (SNF Group 1) or only off-site resources (SNF Group 4) when compared with the SNFs with substantial beds and staffs (SNF Group 2) or PT/OT staff (SNF Group 3). In light of these observations, it is important to recognize that patients may be discharged to a given SNF for reasons other than receiving specialized or high-intensity rehabilitation. These reasons include the 24 h supervised SNF care paid for by Medicare, relief from symptoms and the stress of the illness, and proximity to home [14, 36–40].

Little was previously known about the role of SNF assignment in reducing rehospitalization and mortality across several patient types. Several studies have focused on the association between the SNF and outcomes [5, 8]. For example, no association was found between the risk of readmission or death after 30 days of discharge to a SNF and SNF performance (e.g., staffing rating, inspection rating), but a slight improvement in readmission rates for SNFs with more beds [8]. A lower 30 day readmission risk was found in SNFs with higher online consumer reviews [5]. Other studies have focused on the association between the patient and outcomes; [41, 42] the effectiveness of a SNF discharge; [43] or on specific patient subgroups [44].

A contribution of this paper is the methodological approach. Given the sheer volume of variables (616 in total), the main hurdle was to analyze and summarize the relationship between SNFs, patients, and outcomes in an interpretable and clinically meaningful way. While we opted for dimension reduction via factor analysis, there are alternatives to consider. Patients could be described using established comorbidity indices, [45] such as the Charlson [46]. However, these generally reflect overall comorbidity burden and not diagnoses, and hence, may miss important determinants of the ideal SNF assignment. Only the principal hospital diagnosis could be used, given its singular importance to SNF assignment and relevance to the Hospital Readmissions Reduction Program; however, dimension reduction methods would then be poorly suited, since they depend on variable correlation and principal diagnoses would be largely uncorrelated. With time and resources, relevant stakeholders could derive a small number of groups from the 616 variables. The Patient-Driven Groupings Model [47] by the Centers for Medicare and Medicaid Services could be used, albeit one would still need to work with a large number of patient groups (432 in total). Another option are advanced machine learning approaches (e.g., LASSO), provided their clinical interpretation is not opaque, [48–50] or if a narrower scope is suitable, one could focus on a specific subset of patients [41, 44] or on a reduced set of SNF characteristics, [8] as has been done for prior SNF studies.

Meanwhile, our approach was able to automatically summarize information across numerous highly correlated variables into descriptors. Encouragingly, the descriptors had a reasonable amount of face validity in terms of combining clinically related conditions and diagnoses together (e.g., asthma, respiratory disease not asthma, respiratory disease including asthma, and DRG chronic obstructive pulmonary disease were the strongest indicators of Patient Factor 3). Moreover, the descriptors could have value, not only for the determination of SNF assignment, but for the stratification of rehospitalization risk (i.e. patients with HIV followed by patients with liver disease and congestive heart failure) and mortality risk (i.e. patients with cancer followed by patients with congestive heart failure). Yet, even though the descriptors are, by design, meant to maximize the amount of variation in the patient variables and SNF variables explained, it is possible that they are not clinically relevant to SNF assignment. Thus, the descriptors could also serve as a less overwhelming place to start, than the original 616 variables, for stakeholders to then refine into descriptors or use in conjunction with principal diagnoses to guide SNF assignment. Before study findings can be translated clinically, which would involve characterizing a patient by their factor scores and supporting the patient decision on SNF assignment with predicted mortality and readmission risk of different SNFs, additional work is needed to refine, validate, and replicate findings.

There are several limitations to consider. First, the resulting patient factors were not always statistically relevant. This could be because they are not clinically relevant to SNF assignment (e.g., Patient Factor 12 was marked by female infertility and genitourinary anatomic disorders) or rare (e.g., Patient Factor 5 was marked by HIV). Even when statistically relevant, the patient factors might not be clinically relevant. Patient factor 4, for instance, which is marked by osteoarthritis, had an estimated mortality risk that ranged from 5.0 to 5.3% between SNF groups, a difference that was significant but relatively small. Second, our sample was disproportionately White (89%) compared to the US population, and a majority were female (63%) and residents in three Midwestern states during a limited time period (2010–2017). Consequently, estimated risks may reflect the majority populations in our sample, but generalize poorly to racial and ethnic minorities, to other time periods, or to individuals residing outside of the Midwest. In addition, race, ethnicity, and gender in Medicare claims are not determined based on self-identification and may thus be less valid than their self-identified version [51, 52].

Third, because SNF assignment was not random, our estimates cannot determine whether differences in outcomes can be attributed to the SNF directly or to some other unobserved factors (e.g., distance between patient residence to SNF), which may significantly drive SNF assignment. We suggest using these estimates with caution when deciding what types of SNFs suggest to a patient after hospital discharge. Fourth, the available claims data only contained indicators of rehospitalization and mortality within a 60 day window. A more common window, especially in lieu of the Hospital Readmissions Reduction Program, is 30 days. Additional work is needed to determine whether similar findings can be achieved with 30 day windows. Relatedly, rehospitalization may not always reflect quality of care, given that it may be indicated for many patients regardless of SNF care or that the patient population may differ significantly from one SNF to another. Rehospitalization may even be more likely for some patients in SNFs with attentive staff. Fifth, a claim needed to be submitted for a SNF visit after hospitalization for a patient to be included in the analyzed sample, but the data does not distinguish between long-term residents or community dwelling adults. Thus, with 79% of SNFs in our dataset dually certified as a SNF and a nursing home, the sample may have included both long-term residents and community dwelling adults. Last, SNF variables were determined using the most recent provider service file that was available, which was 2016, and so changes in SNF variables that may have occurred over time are not accounted for in our risk estimation.

## Conclusions

In summary, our work improves our understanding of the risks associated with SNF care among Medicare patients. Our findings support careful consideration of which SNF to assign a patient and add to a growing body of literature suggesting that how patients are managed after discharge in a SNF impacts outcomes. Knowing that certain SNFs are better suited for some patient conditions over others may help providers in deciding where to send their patients after discharge and may help a specific SNF to improve their services for a particular patient population. Our study presents a promising approach for identifying the most suitable SNF for a patient based on their current clinical conditions. However, additional work is needed to refine, validate, and replicate the findings before they can be translated into clinical practice. Once these steps have been taken, we envision that a patient would be assigned their personalized combination of factor scores at a visit based on their current DRG codes and comorbid conditions according to the patient factor model built in this paper. Once this combination is calculated, they can be inputted into the risk prediction models to identify a SNF that is associated with a lower risk of mortality and rehospitalization for patients with similar factor scores. This information could then be used to inform the decision on SNF assignment, taking into account the predicted readmission and mortality risk of different SNFs and the patient’s contextual information.

## Electronic supplementary material

Below is the link to the electronic supplementary material.

Supplementary Material 1: Supporting information is provided in a separate file titled *Additional File 1.docx*. This material consists of details about the GEE model used to estimate rehospitalization and mortality risks (see Additional Section ‘Risks and Odds’). The file also contains unadjusted and adjusted odds of rehospitalization and mortality (Additional Tables A1-A4), unadjusted and adjusted risk of rehospitalization and mortality accounting for the hospital discharge date (Additional Tables A5-A6), accuracy of the logistic regression model (Additional Table A7), VSS scores (Additional Table A8), cross-loaded variables for the SNF and patient factor models (Additional Tables A9 and A10), silhouette scores (Additional Figure A1), DRG grouping versions (Additional Table A11), summary statistics by SNF group (Additional Tables A12-A13), and unadjusted risk of rehospitalization and mortality (Additional Tables A14-A15).

## Data Availability

The datasets generated and/or analyzed during the current study are not publicly available due to privacy and ethical concerns, but are available from the corresponding author on reasonable request and in accordance with institutional policies.

## Abbreviations

AAA: Abdominal aortic aneurysm
DRG: Diagnostic related group
GEE: Generalized estimating equations
HIV: Human immunodeficiency virus
OT: Occupational therapy
PT: Physical therapy
SNF: Skilled nursing facility
VSS: Very simple structure
