# Supplementary material for: Prediction of rehospitalization and mortality risks for skilled nursing facilities using a dimension reduction approach
Source: BMC Geriatr. 2023 Jun 28;23:394. doi: 10.1186/s12877-023-03995-y (PMC10304328; doi:10.1186/s12877-023-03995-y)
Supplement: Supplementary file 1 — Supplementary Material 1: Supporting information is provided in a separate file titled Additional File 1.docx. This material consists of details about the GEE model used to estimate rehospitalization and mortality risks (see Additional Section ‘Risks and Odds’). The file also contains unadjusted and adjusted odds of rehospitalization and mortality (Additional Tables A1-A4), unadjusted and adjusted risk of rehospitalization and mortality accounting for the hospital discharge date (Additional Tables A5-A6), accuracy of the logistic regression model (Additional Table A7), VSS scores (Additional Table A8), cross-loaded variables for the SNF and patient factor models (Additional Tables A9 and A10), silhouette scores (Additional Figure A1), DRG grouping versions (Additional Table A11), summary statistics by SNF group (Additional Tables A12-A13), and unadjusted risk of rehospitalization and mortality (Additional Tables A14-A15). [file 12877_2023_3995_MOESM1_ESM.docx]

**ADDITIONAL FILE 1**

**SUPPORTING INFORMATION**

Contents

[Risks and Odds 2](#_Toc127701368)

[Estimated Odds (Unadjusted and Adjusted) 4](#_Toc127701369)

[Estimated Adjusted Risk with Additional Control (Discharge Date) 8](#_Toc127701370)

[Model Fit Information 10](#_Toc127701371)

[Versions of DRG Grouping 16](#_Toc127701372)

[Summary statistics by SNF group 17](#_Toc127701373)

[Estimated Unadjusted Risk 19](#_Toc127701374)

# **Risks and Odds**

Consider the following visit-level characteristics in the dataset:

- $x$ – covariates
- $s$ – continuous patient-level factor scores
- $a$ – categorical assignment to SNF group
- $y$ – binary outcome

Assume SNF group variable $a$ is encoded as a vector of zeros and ones; a procedure often referred to as dummy coded. As such, $a=e_{i}$ would represent assignment to SNF group $i$, where $e_{i}$ is the standard basis vector. Assume the interaction between SNF group and patient-level factors scores, denoted by $a\times s,$ is also encoded as a vector of mostly zeros, with non-zero values given by $a$. This can be accomplished by reshaping the matrix $a^{'}s$ into a vector.

We use GEE to estimate coefficients ($\iota,\alpha,\beta,\gamma,\tau$) for logistic regression models of the form:

$$\mathrm{logit}\left( P\left( y=1 | x,s,a \right) \right)=\iota+\alpha^{'}x+\beta^{'}s+{\gamma^{'}a+\tau}^{'}\left( a\times s \right):=\eta(x,s,a)$$

We considered models in which we do adjust for covariates $x$ and when we do not adjust for $x$. Unadjusted models assume $\alpha=0$, whereas adjusted models allow for $\alpha$ to take any value.

For either an unadjusted or adjustment model, let $\hat{\eta}(x,s,a)$ capture the above linear predictor function $\eta$ with regression coefficients (i.e., $\iota,\alpha,\beta,\gamma,\tau$) replaced with their estimated values recovered from GEE. Again, the estimated value of $\alpha$ would be zero in the case of an unadjusted model. Estimated odds of the outcome $y=1$ conditional on any combination of covariates $x$, factor scores s, and SNF group $a$ can then recovered by the formula:

$$\exp\hat{\eta}(x,a,s).$$

In this Additional File 1, we report odds for unadjusted and unadjusted models conditional on covariates set to their mean (i.e., $x=0$ since covariates were mean-centered), different SNF assignments (i.e., $a=e_{i}$, $i=1,2,3,4)$ and different combinations of patient-level factors scores (i.e., s=$e_{j}$, $j=1,\ldots,12)$:

$$\mathrm{logit}^{-1}\left( \hat{\eta}(0,e_{i},e_{j}) \right).$$

Note, $s=e_{j}$ corresponds to factor scores that are set to their mean (i.e., zero) except one factor score which is set to +1 its standard deviation (i.e., one).

From fitted regression coefficients, we can also estimate conditional *risks*

$$P\left( y=1 | x,s,a \right)\approx\mathrm{logit}^{-1}\left( \hat{\eta}(x,a,s) \right).$$

In the main text, we report risks if a person *were* assigned to a SNF group, marginalized over covariates and conditional on one of the factor scores being above 1. These risks can be estimated using the formula:

$$E_{n}\left[ \mathrm{logit}^{-1}\left( \hat{\eta}(x,e_{i},s) \right)|s\cdot e_{j}>1 \right]$$

for $i=1,\ldots, 4, j=1,\ldots, 12$ where $E_{n}$ denotes expectation using the empirical distribution. Above, we use $e_{i}$ to model a forced assignment to SNF group $i$. We condition on $s\cdot e_{j}>1$ to capture individuals with patients whose $j$th factor score is greater than 1.

# **Estimated Odds (Unadjusted and Adjusted)**

**Additional Table A1**. Unadjusted odds of rehospitalization (95% confidence intervals) for each patient factor and SNF group.

| **Patient factor** | **SNF group** | | | |  |
| --- | --- | --- | --- | --- | --- |
|  | 1 | 2 | 3 | 4 | *p* |
| **1–Congestive heart failure** | 0.443  0.439-0.447 | 0.441  0.435-0.445 | 0.450  0.445-0.454 | 0.442  0.436-0.447 | 0.01 |
| **2–Non-hematological solid tumor** | 0.381  0.377-0.383 | 0.389  0.384-0.393 | 0.393  0.389-0.396 | 0.381  0.376-0.385 | < 0.001 |
| 3–Asthma | 0.435  0.431-0.438 | 0.436  0.430-0.440 | 0.436  0.431-0.440 | 0.434  0.428-0.438 | 0.90 |
| **4–Osteoarthritis** | 0.318  0.314-0.320 | 0.306  0.302-0.310 | 0.305  0.301-0.308 | 0.331  0.327-0.335 | < 0.001 |
| 5–HIV | 0.366  0.363-0.369 | 0.367  0.363-0.370 | 0.364  0.360-0.367 | 0.365  0.360-0.369 | 0.40 |
| 6–Liver disease | 0.408  0.404-0.410 | 0.409  0.404-0.413 | 0.407  0.402-0.410 | 0.402  0.397-0.406 | 0.10 |
| 7–Chronic skin ulcer | 0.433  0.429-0.436 | 0.435  0.429-0.439 | 0.435  0.430-0.439 | 0.431  0.426-0.436 | 0.61 |
| **8–Depression and depressive disorders** | 0.383  0.379-0.386 | 0.378  0.373-0.382 | 0.384  0.379-0.387 | 0.377  0.372-0.381 | 0.01 |
| **9–Hematological cancer** | 0.390  0.386-0.393 | 0.395  0.390-0.399 | 0.395  0.390-0.398 | 0.388  0.383-0.392 | 0.03 |
| **10–Chronic renal failure** | 0.416  0.412-0.419 | 0.423  0.418-0.428 | 0.425  0.420-0.428 | 0.410  0.405-0.414 | < 0.001 |
| 11–Hyperlipidemia | 0.378  0.375-0.381 | 0.373  0.368-0.377 | 0.375  0.370-0.378 | 0.376  0.371-0.380 | 0.11 |
| 12–Other musculoskeletal including osteoporosis | 0.357  0.353-0.360 | 0.352  0.347-0.355 | 0.356  0.352-0.359 | 0.355  0.350-0.359 | 0.18 |

*Note*. SNF Group 1 = less resources; SNF Group 2 = substantial beds and staff; SNF Group 3 = substantial PT/OT staff; SNF Group 4 = substantial off-site services. Patient factors are bolded if the corresponding *p*-value is less than 0.05 and labeled by a number and the variable that loaded most strongly onto the factor. Odds are a common quantity to report, but can be incomparable between studies due to differences in model specifications and in the sample. For this reason, we have reported estimated risks in the main text and left estimated odds to Additional File 1. *p* values correspond to the same joint Wald hypothesis tests reported in Additional Table 15.

**Additional Table A2**. Adjusted odds of rehospitalization (95% confidence intervals) for each patient factor and SNF cluster.

| **Patient factor** | **SNF group** | | | |  |
| --- | --- | --- | --- | --- | --- |
|  | 1 | 2 | 3 | 4 | *p* |
| 1–Congestive heart failure | 0.440  0.436-0.443 | 0.438  0.433-0.443 | 0.444  0.439-0.448 | 0.439  0.433-0.443 | 0.15 |
| **2–Non-hematological solid tumor** | 0.377  0.374-0.380 | 0.384  0.380-0.388 | 0.388  0.384-0.391 | 0.378  0.373-0.382 | < 0.001 |
| 3–Asthma | 0.422  0.418-0.425 | 0.422  0.417-0.427 | 0.422  0.417-0.426 | 0.421  0.416-0.426 | 0.98 |
| **4–Osteoarthritis** | 0.317  0.314-0.320 | 0.309  0.304-0.312 | 0.306  0.302-0.309 | 0.329  0.324-0.333 | < 0.001 |
| 5–HIV | 0.362  0.358-0.365 | 0.362  0.358-0.365 | 0.359  0.355-0.362 | 0.361  0.356-0.365 | 0.57 |
| 6–Liver disease | 0.396  0.392-0.399 | 0.397  0.393-0.401 | 0.396  0.391-0.399 | 0.391  0.386-0.395 | 0.08 |
| 7–Chronic skin ulcer | 0.415  0.411-0.418 | 0.413  0.408-0.417 | 0.416  0.411-0.419 | 0.413  0.407-0.417 | 0.66 |
| **8–Depression and depressive disorders** | 0.373  0.369-0.375 | 0.370  0.365-0.374 | 0.376  0.371-0.379 | 0.366  0.361-0.370 | 0.004 |
| 9–Hematological cancer | 0.387  0.383-0.390 | 0.392  0.387-0.396 | 0.391  0.387-0.394 | 0.386  0.381-0.390 | 0.07 |
| **10–Chronic renal failure** | 0.406  0.402-0.409 | 0.411  0.405-0.415 | 0.414  0.409-0.417 | 0.403  0.398-0.408 | 0.001 |
| 11–Hyperlipidemia | 0.381  0.377-0.383 | 0.377  0.372-0.381 | 0.377  0.372-0.380 | 0.378  0.373-0.382 | 0.24 |
| 12–Other musculoskeletal including osteoporosis | 0.372  0.369-0.375 | 0.369  0.364-0.373 | 0.371  0.367-0.375 | 0.370  0.365-0.374 | 0.48 |

*Note.* SNF Group 1 = less resources; SNF Group 2 = substantial beds and staff; SNF Group 3 = substantial PT/OT staff; SNF Group 4 = substantial off-site services. Patient factors are bolded if the corresponding *p*-value is less than 0.05 and labeled by a number and the variable that loaded most strongly onto the factor. Odds are a common quantity to report, but can be incomparable between studies due to differences in model specifications and in the sample. For this reason, we have reported estimated risks in the main text and left estimated odds to Additional File 1. *p* values correspond to the same joint Wald hypothesis tests reported in reported in Additional Table 16.

**Additional Table A3**. Unadjusted odds of mortality (95% confidence intervals) for each patient factor and SNF group.

| **Patient factor** | **SNF group** | | | |  |
| --- | --- | --- | --- | --- | --- |
|  | 1 | 2 | 3 | 4 | *p* |
| **1–Congestive heart failure** | 0.173  0.171-0.175 | 0.164  0.161-0.167 | 0.168  0.165-0.170 | 0.172  0.169-0.174 | < 0.001 |
| 2–Non-hematological solid tumor | 0.171  0.169-0.173 | 0.172  0.169-0.173 | 0.173  0.171-0.175 | 0.172  0.169-0.174 | 0.41 |
| 3–Asthma | 0.163  0.161-0.164 | 0.162  0.159-0.164 | 0.162  0.159-0.164 | 0.162  0.159-0.164 | 0.90 |
| **4–Osteoarthritis** | 0.073  0.072-0.074 | 0.076  0.074-0.077 | 0.076  0.074-0.077 | 0.073  0.071-0.074 | < 0.001 |
| 5–HIV | 0.134  0.132-0.136 | 0.134  0.132-0.135 | 0.135  0.133-0.137 | 0.135  0.132-0.137 | 0.71 |
| 6–Liver disease | 0.152  0.150-0.153 | 0.151  0.148-0.153 | 0.151  0.149-0.153 | 0.151  0.148-0.153 | 0.57 |
| **7–Chronic skin ulcer** | 0.152  0.150-0.154 | 0.159  0.156-0.160 | 0.153  0.151-0.155 | 0.155  0.152-0.157 | < 0.001 |
| **8–Depression and depressive disorders** | 0.134  0.131-0.135 | 0.129  0.127-0.131 | 0.133  0.131-0.135 | 0.133  0.130-0.135 | 0.006 |
| 9–Hematological cancer | 0.155  0.152-0.156 | 0.155  0.152-0.156 | 0.155  0.152-0.156 | 0.155  0.153-0.157 | 0.92 |
| 10–Chronic renal failure | 0.145  0.142-0.146 | 0.147  0.144-0.149 | 0.145  0.143-0.147 | 0.145  0.142-0.147 | 0.29 |
| 11–Hyperlipidemia | 0.128  0.126-0.129 | 0.130  0.127-0.131 | 0.128  0.126-0.129 | 0.128  0.125-0.129 | 0.41 |
| 12–Other musculoskeletal including osteoporosis | 0.129  0.127-0.130 | 0.130 0.127-0.131 | 0.129  0.127-0.131 | 0.128  0.126-0.130 | 0.85 |

*Note.* SNF Group 1 = less resources; SNF Group 2 = substantial beds and staff; SNF Group 3 = substantial PT/OT staff; SNF Group 4 = substantial off-site services. Patient factors are bolded if the corresponding *p*-value is less than 0.05 and labeled by a number and the variable that loaded most strongly onto the factor. Odds are a common quantity to report, but can be incomparable between studies due to differences in model specifications and in the sample. For this reason, we have reported estimated risks in the main text and left estimated odds to Additional File 1. *p* values correspond to the same joint Wald hypothesis tests reported in reported in Table 3.

**Additional Table A4**. Adjusted odds of mortality (95% Confidence intervals) for each patient factor and SNF group.

| **Patient factor** | **SNF group** | | | |  |
| --- | --- | --- | --- | --- | --- |
|  | 1 | 2 | 3 | 4 | *p* |
| **1–Congestive heart failure** | 0.152  0.150-0.153 | 0.144  0.141-0.146 | 0.147  0.145-0.149 | 0.151  0.148-0.153 | < 0.001 |
| 2–Non-hematological solid tumor | 0.156  0.154-0.157 | 0.156  0.154-0.158 | 0.158  0.155-0.160 | 0.157  0.154-0.159 | 0.40 |
| 3–Asthma | 0.156  0.153-0.157 | 0.154  0.151-0.156 | 0.153  0.151-0.155 | 0.156  0.153-0.158 | 0.28 |
| **4–Osteoarthritis** | 0.077  0.075-0.077 | 0.079  0.077-0.080 | 0.080  0.078-0.081 | 0.076  0.074-0.077 | < 0.001 |
| 5–HIV | 0.126  0.123-0.127 | 0.126  0.124-0.127 | 0.127  0.125-0.128 | 0.127  0.124-0.129 | 0.73 |
| 6–Liver disease | 0.145  0.143-0.146 | 0.143  0.141-0.145 | 0.143  0.141-0.145 | 0.145  0.142-0.146 | 0.29 |
| **7–Chronic skin ulcer** | 0.142  0.140-0.144 | 0.147  0.145-0.149 | 0.143  0.140-0.144 | 0.145  0.142-0.147 | < 0.001 |
| **8–Depression and depressive disorders** | 0.131  0.129-0.132 | 0.127  0.124-0.129 | 0.129  0.127-0.131 | 0.131  0.128-0.133 | 0.007 |
| 9–Hematological cancer | 0.142  0.140-0.143 | 0.142  0.139-0.143 | 0.141  0.139-0.143 | 0.143  0.140-0.144 | 0.82 |
| 10–Chronic renal failure | 0.137  0.135-0.138 | 0.139  0.137-0.141 | 0.137  0.135-0.139 | 0.138  0.135-0.139 | 0.21 |
| 11–Hyperlipidemia | 0.112  0.110-0.113 | 0.113  0.111-0.115 | 0.113  0.111-0.114 | 0.112  0.110-0.114 | 0.81 |
| 12–Other musculoskeletal including osteoporosis | 0.119  0.117-0.120 | 0.119  0.117-0.121 | 0.120 0.118-0.121 | 0.118  0.116-0.120 | 0.63 |

*Note.* SNF Group 1 = less resources; SNF Group 2 = substantial beds and staff; SNF Group 3 = substantial PT/OT staff; SNF Group 4 = substantial off-site services. Patient factors are bolded if the corresponding *p*-value is less than 0.05 and labeled by a number and the variable that loaded most strongly onto the factor. Odds are a common quantity to report, but can be incomparable between studies due to differences in model specifications and in the sample. For this reason, we have reported estimated risks in the main text and left estimated odds to Additional File 1. *p* values correspond to the same joint Wald hypothesis tests reported in reported in Table 4.

# **Estimated Adjusted Risk with Additional Control (Discharge Date)**

**Additional Table A5**. Adjusted risk, in percentages, of rehospitalization (95% confidence intervals) for each patient factor and SNF cluster after adjusting for the hospital discharge date.

| **Patient factor** | **SNF group** | | | |  | |
| --- | --- | --- | --- | --- | --- | --- |
|  | 1 | 2 | 3 | 4 | *p* |  |
| 1–Congestive heart failure | 36.7  36.3-37.0 | 36.6  36.1-37.0 | 37.2  36.8-37.5 | 36.4  36.0-36.9 | 0.15 |  |
| **2–Non-hematological solid tumor** | 31.1  30.7-31.5 | 31.9  31.4-32.5 | 32.5  32.0-32.9 | 31.3  30.7-31.9 | < 0.001 |  |
| 3–Asthma | 33.9  33.6-34.2 | 33.9  33.4-34.3 | 33.8  33.4-34.2 | 34.0  33.5-34.4 | 0.98 |  |
| **4–Osteoarthritis** | 22.6  22.4-22.9 | 21.9  21.6-22.2 | 21.7  21.4-22.0 | 23.5  23.2-23.9 | < 0.001 |  |
| 5–HIV | 39.9  35.2-44.5 | 39.2  34.7-43.7 | 35.4  30.8-39.9 | 38.0  31.1-44.9 | 0.58 |  |
| 6–Liver disease | 38.4  37.8-39.0 | 38.4  37.5-39.3 | 38.1  37.3-38.9 | 37.4  36.4-38.3 | 0.08 |  |
| 7–Chronic skin ulcer | 36.0  35.7-36.3 | 35.6  35.2-36.1 | 36.0  35.6-36.4 | 35.7  35.2-36.2 | 0.65 |  |
| **8–Depression and depressive disorders** | 28.4  28.1-28.6 | 27.9  27.5-28.3 | 28.5  28.1-28.8 | 27.8  27.4-28.2 | 0.004 |  |
| 9–Hematological cancer | 35.3  34.4-36.2 | 36.6  35.3-37.9 | 36.2  35.1-37.3 | 35.5  34.1-36.8 | 0.07 |  |
| **10–Chronic renal failure** | 35.5  35.2-35.7 | 35.7  35.3-36.1 | 36.1  35.7-36.4 | 35.1  34.7-35.5 | 0.001 |  |
| 11–Hyperlipidemia | 31.6  31.3-31.8 | 31.3  30.9-31.6 | 31.4  31.1-31.7 | 31.3  30.9-31.6 | 0.24 |  |
| 12–Other musculoskeletal including osteoporosis | 27.7  27.5-28.0 | 27.2  26.8-27.6 | 27.5  27.2-27.8 | 27.6  27.2-28.0 | 0.48 |  |

*Note*. SNF Group 1 = less resources; SNF Group 2 = substantial beds and staff; SNF Group 3 = substantial PT/OT staff; SNF Group 4 = substantial off-site services. Patient factors are bolded if the corresponding *p*-value is less than 0.05 and labeled by a number and the variable that loaded most strongly onto the factor. These estimates were obtained using the exact procedure as was used to get estimates reported in main text, except that discharge date (mean-centered year and month of discharge data) was added as a covariate to the regression model. Estimates are negligibly different from those reported in main text, suggesting that discharge date is not an important confound.

**Additional Table A6**. Adjusted risk, in percentages, of mortality (95% confidence intervals) for each patient factor and SNF cluster after adjusting for the hospital discharge date.

| **Patient factor** | **SNF group** | | | |  |
| --- | --- | --- | --- | --- | --- |
|  | 1 | 2 | 3 | 4 | *p* |
| **1–Congestive heart failure** | 20.2  19.9-20.5 | 18.8  18.5-19.2 | 19.2  18.9-19.5 | 20.2  19.8-20.6 | < 0.001 |
| 2–Non-hematological solid tumor | 20.9  20.5-21.2 | 20.7  20.2-21.3 | 21.1  20.7-21.5 | 21.3  20.7-21.8 | 0.39 |
| 3–Asthma | 17.0  16.8-17.3 | 16.7  16.3-17.1 | 16.6  16.2-16.9 | 17.0  16.7-17.4 | 0.25 |
| **4–Osteoarthritis** | 5.0  4.9-5.1 | 5.2  5.1-5.3 | 5.3  5.2-5.4 | 5.0  4.9-5.1 | < 0.001 |
| 5–HIV | 11.4  8.1-14.6 | 11.6  8.4-14.8 | 13.7  10.2-17.2 | 13.2  7.9-18.5 | 0.74 |
| 6–Liver disease | 17.8  17.3-18.3 | 17.1  16.4-17.9 | 17.2  16.6-17.8 | 17.7  16.9-18.5 | 0.29 |
| **7–Chronic skin ulcer** | 15.6  15.4-15.9 | 16.4  16.0-16.7 | 15.5  15.2-15.8 | 16.2  15.8-16.6 | < 0.001 |
| **8–Depression and depressive disorders** | 11.1  10.9-11.3 | 10.6  10.4-10.9 | 10.9  10.6-11.1 | 11.2  10.9-11.5 | 0.007 |
| 9–Hematological cancer | 21.6  20.7-22.4 | 21.2  20.0-22.4 | 21.2  20.2-22.3 | 22.1  20.8-23.4 | 0.81 |
| 10–Chronic renal failure | 15.8  15.5-16.0 | 15.9  15.6-16.2 | 15.5  15.2-15.8 | 16.0  15.6-16.3 | 0.20 |
| 11–Hyperlipidemia | 13.8  13.6-14.0 | 13.7  13.5-14.0 | 13.6  13.3-13.8 | 13.9  13.6-14.2 | 0.78 |
| 12–Other musculoskeletal including osteoporosis | 11.2  11.1-11.4 | 11.2  11.0-11.5 | 11.3  11.1-11.5 | 11.2  11.0-11.5 | 0.61 |

*Note*. SNF Group 1 = less resources; SNF Group 2 = substantial beds and staff; SNF Group 3 = substantial PT/OT staff; SNF Group 4 = substantial off-site services. Patient factors are bolded if the corresponding *p*-value is less than 0.05 and labeled by a number and the variable that loaded most strongly onto the factor. These estimates were obtained using the exact procedure as was used to get estimates reported in main text, except that discharge date (mean-centered year and month of discharge data) was added as a covariate to the regression model. Estimates are negligibly different from those reported in main text, suggesting that discharge date is not an important confound.

# **Model Fit Information**

**Additional Table A7**. Average accuracy and area under the receiver operating characteristic (ROC) curve for 10-folds cross validation iterations. Accuracy at each iteration was calculated using a 50% threshold.

| **Outcome** | **Model** | **Average**  **Accuracy** | **Average**  **Area under the ROC curve** |
| --- | --- | --- | --- |
| Rehospitalization | Unadjusted | 0.69 | 0.63 |
|  | Adjusted | 0.71 | 0.66 |
| Mortality | Unadjusted | 0.86 | 0.68 |
|  | Adjusted | 0.88 | 0.71 |

*Note.* These estimates were obtained using K-fold cross validation to assess the accuracy of our logistic regression models. To this end, we split the entire sample into 10 disjoint folds. For each fold, we trained our models of rehospitalization and mortality within 60 days using all the data excluding SNF visits from the current/fixed fold. In each of the ten iterations, we built the ROC curve using the excluded fold as the validation set. We repeated these steps until all of the folds are used as validation sets. We report the average area under the curve for the 10 iterations. We also report average accuracy for the 10 iterations when using a 50% predicted probability as the threshold for specifying the binary prediction.

**Additional Table A8**. Very Simple Structure (VSS) score for patient and SNF factor analysis.

|  | **VSS** | |
| --- | --- | --- |
| **Number of factors** | **Patient model** | **SNF model** |
| 1 | 0.029 | 0.29 |
| 2 | 0.041 | 0.40 |
| 3 | 0.047 | 0.47 |
| 4 | 0.053 | 0.45 |
| 5 | 0.053 | 0.47 |
| 6 | 0.055 | 0.42 |
| 7 | 0.058 | 0.45 |
| 8 | 0.058 | 0.46 |
| 9 | 0.062 | 0.48 |
| 10 | 0.061 | 0.47 |
| 11 | 0.063 | 0.48 |
| 12 | 0.064 | 0.46 |
| 13 | 0.063 | 0.49 |
| 14 | 0.065 | 0.48 |
| 15 | 0.066 | 0.47 |
| 16 | 0.069 | 0.48 |
| 17 | 0.069 | 0.48 |
| 18 | 0.071 | 0.47 |
| 19 | 0.071 | 0.48 |
| 20 | 0.071 | 0.47 |

*Note.* In addition to silhouette scores, VSS scores are a measure of model fit and helped guide the choice in the number of factors. Larger VSS scores are generally preferred.

**Additional Table A9**. Cross loaded clinical variables (patient variables with a loading exceeding 0.2 for two or more factors).

| **Clinical**  **Variable** | **Cross Loaded**  **Factors** |
| --- | --- |
| Coronary atherosclerosis | P1 – Congestive heart failure  P11 - Hyperlipidemia |
| Chronic renal failure | P1- Congestive heart failure  P10 - Chronic renal failure |
| Malignant neoplasm | P2 - Non-hematological solid tumor  P9 - Hematological cancer |
| Central/peripheral nervous system disorders | P4 - Osteoarthritis  P7- Chronic skin ulcer  P8 - Depression and depressive disorders |
| Hyperlipidemia | P4 - Osteoarthritis  P10 - Chronic renal failure  P11- Hyperlipidemia |
| Obesity | P4 - Osteoarthritis  P10 - Chronic renal failure |
| Peripheral atherosclerosis | P7 - Chronic skin ulcer  P11- Hyperlipidemia |
| Hypertension | P4 - Osteoarthritis  P10 - Chronic renal failure  P11 - Hyperlipidemia |

*Note.* Cross-loadings are reported to point out that clinical variables could be indicators of more than one patient factor. For example, obesity is an indicator of both the “Osteoarthritis” factor as well as the “Chronic renal failure” factor. Patient factors are labeled by a number and the variable that loaded most strongly onto the factor.

**Additional Table A10**. Cross loaded SNF variables (SNF variables with a loading exceeding 0.2 for two or more factors).

| **SNF**  **Variable** | **Cross Loaded**  **Factors** |
| --- | --- |
| Occupational therapists - full time | C1 – Number of beds and staff  C3 – PT/OT capacity |
| Occupational therapy assistants - full time | C1 – Number of beds and staff  C3 – PT/OT capacity |
| Physical therapists - full time | C1 – Number of beds and staff  C3 – PT/OT capacity |
| Physical therapy aides - full time | C1 – Number of beds and staff  C3 – PT/OT capacity |
| Physical therapy assistants - full time | C1 – Number of beds and staff  C3 – PT/OT capacity |
| Speech pathologists - full time | C1 – Number of beds and staff  C3 – PT/OT capacity |

*Note.* Cross-loadings are reported to point out that SNF variables could be indicators of more than one SNF factor. For example, the number of full time occupational therapists is an indicator of both the “Numbers of beds and staffs” factor as well as the “PT/OT capacity” factor.

**Additional Figure A1**. Silhouette score per each factor model and number of SNF clusters.


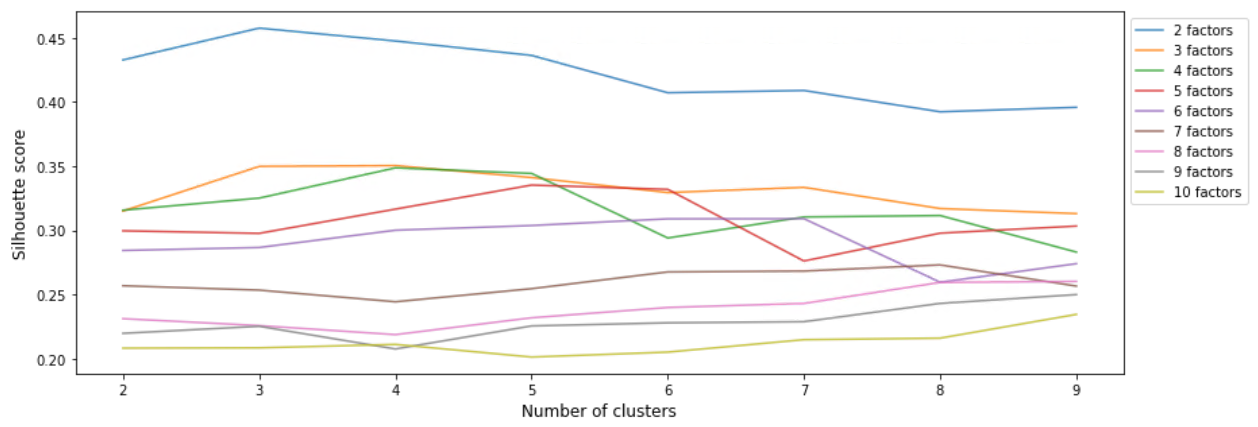


# **Versions of DRG Grouping**

**Additional Table A11**. DRG grouper versions with effective dates.

| **MS-DRG**  **Grouper Version** | **Effective**  **Date Range** |
| --- | --- |
| MS-DRG 35.0 | 10/01/2017-09/30/2018 |
| MS-DRG 34.0 | 10/01/2016-09/30/2017 |
| MS-DRG 33.0 | 10/01/2015-09/30/2016 |
| MS-DRG 32.0 | 10/01/2014-09/30/2015 |
| MS-DRG 31.0 | 10/01/2013-09/30/2014 |
| MS-DRG 30.0 | 10/01/2012-09/30/2013 |
| MS-DRG 29.0 | 10/01/2011-09/30/2012 |
| MS-DRG 28.0 | 10/01/2010-09/30/2011 |
| MS-DRG 27.0 | 10/01/2009-09/30/2010 |
| MS-DRG 26.0 | 10/01/2008-09/30/2009 |
| MS-DRG 25.0 | 10/01/2007-09/30/2008 |

# **Summary statistics by SNF group**

**Additional Table A12**. Sample statistics of claims (n = 1,060,337) by SNF group.

| **Variable name** | | **SNF Group** | | | | |  |  |  |  |
| --- | --- | --- | --- | --- | --- | --- | --- | --- | --- | --- |
|  |  | 1 | 2 | 3 | 4 |  |  |  |  |  |
| **Outcomes** |  |  |  |  |  |  |  |  |  |  |
| Death within 60 days of  hospital discharge, n (%) | | 55573 (13.3) | 26689 (13.1) | 31789 (12.1) | 25401 (14.2) |  |  |  |  |  |
| 60 day rehospitalization, n (%) | | 123000 (29.4) | 65419 (32.2) | 80680 (30.8) | 51627 (28.8) |  |  |  |  |  |
| **Patient variables** |  |  |  |  |  |  |  |  |  |  |
| Hospital length of stay in days, mean (SD) | | 6.1 (4.9) | 6.5 (5.3) | 6.3 (5.1) | 6.1 (4.8) |  |  |  |  |  |
| Age in years, mean (SD) | | 78.5 (9.9) | 78.3 (10.1) | 78.5 (9.5) | 78.4 (10.1) |  |  |  |  |  |
| Female indicator, n (%) | | 262919 (62.9) | 125516 (61.9) | 165044 (63.1) | 110888 (62.0) |  |  |  |  |  |
| Complicated, n (%) | | 265678 (63.5) | 132373 (65.3) | 166220 (63.6) | 114708 (64.1) |  |  |  |  |  |
| Race/ethnicity, n (%) | |  |  |  |  |  |  |  |  |  |
| - Asian/Pacific islander | | 1895 (0.4) | 1865 (0.9) | 2067 (0.8) | 418 (0.2) |  |  |  |  |  |
| - Black | | 26767 (6.4) | 28610 (14.1) | 25636 (9.8) | 8351 (4.7) |  |  |  |  |  |
| - Hispanic | | 2882 (0.7) | 2454 (1.2) | 2589 (1.0) | 807 (0.4) |  |  |  |  |  |
| - Native American | | 1412 (0.3) | 238 (0.1) | 373 (0.1) | 976 (0.5) |  |  |  |  |  |
| - Other | | 2430 (0.5) | 1885 (0.9) | 2171 (0.8) | 640 (0.3) |  |  |  |  |  |
| - Unknown | | 1109 (0.2) | 589 (0.3) | 832 (0.3) | 444 (0.2) |  |  |  |  |  |
| - White | | 381298 (91.2) | 167057 (82.4) | 227500 (87.1) | 167042 (93.4) |  |  |  |  |  |
| Comorbidities (top 5), n (%) | |  |  |  |  |  |  |  |  |  |
| - Hypertension | | 294075 (70.3) | 144424 (71.2) | 196318 (75.1) | 119607 (66.9) |  | | | | |
| - Hyperlipidemia | | 195664 (46.8) | 95226 (46.9) | 134157 (51.36) | 77288 (43.2) |  | | | |  |
| - Osteoarthritis | | 145666 (34.8) | 75270 (37.1) | 104271 (39.9) | 54485 (30.4) |  | | |  |  |
| - Diabetes mellitus | | 136631 (32.7) | 67840 (33.4) | 89349 (34.2) | 58570 (32.7) |  | | | | |
| - Conduction disorder or cardiac dysrhythmia | | 121959 (29.1) | 57552 (28.3) | 80767 (30.9) | 49679 (27.8) |  | |  |  |  |
| DRGs (top 5), n (%) | |  |  |  |  |  |  |  |  |  |
| - Major hip and knee joint replacement or Reattachment of lower extremity | | 36664 (8.7) | 15058 (7.4) | 25282 (9.6) | 12825 (7.1) |  |  |  |  |  |
| - Septicemia or severe sepsis | | 32360 (7.7) | 15593 (7.6) | 18123 (6.9) | 13619 (7.6) |  |  |  |  |  |
| - Hip and femur procedures except major joint | | 18981 (4.5) | 7916 (3.9) | 10931 (4.1) | 8284 (4.6) |  |  |  |  |  |
| - Simple pneumonia and pleurisy | | 16698 (3.9) | 6952 (3.4) | 7714 (2.9) | 8549 (4.7) |  |  |  |  |  |
| - Kidney and urinary tract infections | | 14837 (3.5) | 7780 (3.8) | 8746 (3.3) | 6636 (3.7) |  |  |  |  |  |

*Note*. SNF Group 1 = less resources; SNF Group 2 = substantial beds and staff; SNF Group 3 = substantial PT/OT staff; SNF Group 4 = substantial off-site services.

**Additional Table A13**. Sample statistics of SNFs (n = 13,708) by SNF group.

| **Variable name** | **SNF Group** | | | |
| --- | --- | --- | --- | --- |
|  | 1 | 2 | 3 | 4 |
| Number of beds (top 5), mean (SD) |  |  |  |  |
| - Total number of beds | 88.2 (36.7) | 194.2 (82.5) | 146.4 (60.8) | 94.9 (40.3) |
| - Medicare/Medicaid beds | 85.9 (34.8) | 192.0 (80.8) | 144.8 (60.2) | 92.9 (38.9) |
| - Dually certified beds | 77.1 (40.2) | 180.1 (86.3) | 133.2 (68.3) | 87.2 (41.8) |
| - Number of beds – Alzheimer | 3.2 (10.3) | 12.3 (25.6) | 6.1 (15.7) | 4.1 (10.5) |
| - Number of beds – Rehabilitation | 0.3 (3.6) | 2.0 (10.6) | 2.1 (12.0) | 0.47 (3.8) |
| Staff count (top 5), mean (SD) |  |  |  |  |
| - Certified nurse aides - full time | 24.9 (14.1) | 63.5 (40.8) | 45.9 (28.2) | 26.1 (16.5) |
| - Licensed practical/vocational nurses - full time | 9.8 (8.0) | 22.6 (17.6) | 18.1 (20.0) | 9.8 (7.3) |
| - Food service personnel - full time | 7.1 (5.2) | 14.7 (15.1) | 11.9 (8.9) | 7.5 (5.7) |
| - Certified nurse aides - part time | 5.1 (5.9) | 13.5 (18.3) | 11.1 (12.1) | 5.7 (7.2) |
| - Housekeeping personnel - full time | 5.1 (4.4) | 13.5 (24.8) | 10.1 (9.7) | 5.4 (4.9) |
| Services (top 5), n (%) |  |  |  |  |
| - Nursing services on-site | 7053.0 (100.0) | 1626.0 (99.9) | 2174.0 (100.0) | 2827.0 (99.1) |
| - Dietary services on-site | 7050.0 (100.0) | 1625.0 (99.9) | 2172.0 (99.9) | 2814.0 (98.6) |
| - Housekeeping services on-site | 7036.0 (99.8) | 1624.0 (99.8) | 2167.0 (99.7) | 2803.0 (98.2) |
| - Physical Therapy on-site | 7050.0 (100.0) | 1626.0 (99.9) | 2173.0 (100.0) | 2755.0 (96.5) |
| - Occupational therapy on-site | 7046.0 (99.9) | 1627.0 (100.0) | 2174.0 (100.0) | 2741.0 (96.0) |
| Provider type, n (%) |  |  |  |  |
| - Skilled Nursing Facility/Nursing Facility (Dually Certified) | 5342 (75.7) | 1364 (83.4) | 1733 (79.7) | 2372 (83.1) |
| - Skilled Nursing Facility/Nursing Facility (Distinct Part) | 1112 (15.7) | 249 (15.3) | 332 (15.2) | 402 (14.0) |
| - Skilled Nursing Facility | 599 (8.4) | 14 (0.8) | 109 (5.0) | 80 (2.8) |
| Urban indicator, n (%) |  |  |  |  |
| - Urban | 5065 (71.8) | 1515 (93.1) | 2024 (93.1) | 1507 (52.8) |
| - Rural | 1988 (28.2) | 112 (6.9) | 150 (6.9) | 1347 (47.1) |

*Note*. SNF Group 1 = less resources; SNF Group 2 = substantial beds and staff; SNF Group 3 = substantial PT/OT staff; SNF Group 4 = substantial off-site services.

# **Estimated Unadjusted Risk**

**Additional Table A14**. Unadjusted risk, in percentages, of rehospitalization (95% confidence intervals) for each patient factor and SNF cluster.

| **Factor** | **SNF group** | | | |  |
| --- | --- | --- | --- | --- | --- |
|  | 1 | 2 | 3 | 4 | *p* |
| **1–Congestive heart failure** | 36.2  35.9-36.5 | 36.1  35.7-36.6 | 37.0  36.6-37.4 | 35.9  35.5-36.4 | 0.01 |
| **2–Non-hematological solid tumor** | 30.8  30.4-31.2 | 31.7  31.2-32.3 | 32.4  31.9-32.9 | 31.0  30.4-31.6 | < 0.001 |
| 3–Asthma | 33.6  33.3-34.0 | 33.5  33.1-34.0 | 33.6  33.2-34.0 | 33.6  33.2-34.1 | 0.90 |
| **4–Osteoarthritis** | 22.8  22.5-23.0 | 21.8  21.5-22.1 | 21.8  21.5-22.0 | 23.9  23.5-24.2 | < 0.001 |
| 5–HIV | 38.8  34.2-43.4 | 39.1  34.5-43.7 | 34.0  29.4-38.6 | 37.2  30.4-44.1 | 0.40 |
| 6–Liver disease | 38.1  37.4-38.7 | 38.0  37.1-38.8 | 37.7  36.9-38.4 | 37.2  36.2-38.1 | 0.10 |
| 7–Chronic skin ulcer | 35.5  35.2-35.8 | 35.5  35.0-36.0 | 35.8  35.3-36.2 | 35.3  34.8-35.8 | 0.61 |
| **8–Depression and depressive disorders** | 28.1  27.9-28.4 | 27.4  27.0-27.8 | 28.1  27.7-28.4 | 27.7  27.2-28.1 | 0.01 |
| **9–Hematological cancer** | 34.7  33.8-35.6 | 35.9  34.5-37.2 | 35.9  34.8-37.0 | 34.7  33.3-36.1 | 0.03 |
| **10–Chronic renal failure** | 34.7  34.4-35.0 | 35.2  34.8-35.6 | 35.5  35.2-35.9 | 34.2  33.7-34.6 | < 0.001 |
| 11–Hyperlipidemia | 31.1  30.8-31.3 | 30.7  30.3-31.0 | 31.0  30.7-31.3 | 30.8  30.4-31.2 | 0.11 |
| 12–Other musculoskeletal including osteoporosis | 27.3  27.0-27.5 | 26.5  26.2-26.9 | 27.0  26.7-27.3 | 27.2  26.8-27.6 | 0.18 |

*Note.* SNF Group 1 = less resources; SNF Group 2 = substantial beds and staff; SNF Group 3 = substantial PT/OT staff; SNF Group 4 = substantial off-site services. Patient factors are bolded if the corresponding *p*-value is less than 0.05 and labeled by a number and the variable that loaded most strongly onto the factor.

**Additional Table A15**. Unadjusted risk of mortality (95% confidence intervals) for each patient factor and SNF cluster.

| **Factor** | **SNF group** | | | |  |
| --- | --- | --- | --- | --- | --- |
|  | 1 | 2 | 3 | 4 | *p* |
| **1–Congestive heart failure** | 20.4  20.1-20.7 | 19.1  18.7-19.4 | 19.4  19.1-19.7 | 20.2  19.8-20.6 | < 0.001 |
| 2–Non-hematological solid tumor | 20.9  20.5-21.2 | 20.8  20.3-21.3 | 21.2  20.7-21.6 | 21.1  20.5-21.7 | 0.41 |
| 3–Asthma | 17.1  16.8-17.3 | 16.9  16.6-17.3 | 16.8  16.5-17.1 | 16.9  16.6-17.3 | 0.90 |
| **4–Osteoarthritis** | 5.2  5.1-5.3 | 5.4  5.3-5.6 | 5.5  5.3-5.6 | 5.2  5.1-5.4 | < 0.001 |
| 5–HIV | 12.3  8.9-15.7 | 11.6  8.4-14.7 | 14.2  10.6-17.7 | 13.5  8.1-19.0 | 0.71 |
| 6–Liver disease | 18.2  17.7-18.8 | 17.7  17.0-18.5 | 17.9  17.2-18.5 | 17.8  17.0-18.6 | 0.57 |
| **7–Chronic skin ulcer** | 15.8  15.6-16.1 | 16.7  16.4-17.1 | 15.8  15.5-16.2 | 16.2  15.8-16.6 | < 0.001 |
| **8–Depression and depressive disorders** | 11.4  11.2-11.6 | 10.9  10.6-11.2 | 11.3  11.1-11.6 | 11.3  11.0-11.6 | 0.006 |
| 9–Hematological cancer | 21.6  20.8-22.4 | 21.4  20.2-22.7 | 21.4  20.4-22.5 | 22.1  20.8-23.4 | 0.92 |
| 10–Chronic renal failure | 16.0  15.8-16.3 | 16.2  15.9-16.5 | 15.8  15.5-16.1 | 16.1  15.7-16.4 | 0.29 |
| 11–Hyperlipidemia | 13.9  13.7-14.1 | 14.0  13.8-14.3 | 13.8  13.5-14.0 | 13.9  13.6-14.2 | 0.41 |
| 12–Other musculoskeletal including osteoporosis | 11.3  11.1-11.5 | 11.3  11.1-11.6 | 11.3  11.1-11.5 | 11.2  10.9-11.5 | 0.85 |

*Note.* SNF Group 1 = less resources; SNF Group 2 = substantial beds and staff; SNF Group 3 = substantial PT/OT staff; SNF Group 4 = substantial off-site services. Patient factors are bolded if the corresponding *p*-value is less than 0.05 and labeled by a number and the variable that loaded most strongly onto the factor.
